# Changes in Stress-Mediated Markers in a Human Cardiomyocyte Cell Line under Hyperglycemia

**DOI:** 10.3390/ijms221910802

**Published:** 2021-10-07

**Authors:** Vikram Thakur, Narah Alcoreza, Jasmine Cazares, Munmun Chattopadhyay

**Affiliations:** 1 Center of Emphasis in Diabetes and Metabolism, Department of Molecular and Translational Medicine, Paul L. Foster School of Medicine, Texas Tech University Health Sciences Center El Paso, El Paso, TX 79905, USA; vikram.thakur@ttuhsc.edu; 2 Graduate School of Biomedical Sciences, Texas Tech University Health Sciences Center El Paso, El Paso, TX 79905, USA; nalcorez@gmail.com (N.A.); jascaz05@gmail.com (J.C.)

**Keywords:** hyperglycemia, cardiomyocyte, Connexin 43, inflammation, oxidative stress

## Abstract

Diabetes is a major risk factor for cardiovascular diseases, especially cardiomyopathy, a condition in which the smooth muscles of the heart become thick and rigid, affecting the functioning of cardiomyocytes, the contractile cells of the heart. Uncontrolled elevated glucose levels over time can result in oxidative stress, which could lead to inflammation and altered epigenetic mechanisms. In the current study, we investigated whether hyperglycemia can modify cardiac function by directly affecting these changes in cardiomyocytes. To evaluate the adverse effect of high glucose, we measured the levels of gap junction protein, connexin 43, which is responsible for modulating cardiac electric activities and Troponin I, a part of the troponin complex in the heart muscles, commonly used as cardiac markers of ischemic heart disease. AC16 human cardiomyocyte cells were used in this study. Under hyperglycemic conditions, these cells demonstrated altered levels of connexin 43 and Troponin-I after 24 h of exposure. We also examined hyperglycemia induced changes in epigenetic markers: H3K9me1, Sirtuin-1 (SIRT1), and histone deacetylase (HDAC)-2 as well as in inflammatory and stress-related mediators, such as heat shock protein (HSP)-60, receptor for advanced glycation end products (RAGE), toll-like receptor (TLR)-4, high mobility group box (HMGB)-1 and CXC chemokine receptor (CXCR)-4. Cardiomyocytes exposed to 25mM glucose resulted in the downregulation of HSP60 and SIRT1 after 48 h. We further examined that hyperglycemia mediated the decrease in the gap junction protein CX43, as well as CXC chemokine receptor CXCR4 which may affect the physiological functions of the cardiomyocytes when exposed to high glucose for 24 and 48 h. Upregulated expression of DNA-binding nuclear protein HMGB1, along with changes in histone methylation marker H3K9me1 have demonstrated hyperglycemia-induced damage to cardiomyocyte at 24 h of exposure. Our study established that 24 to 48 h of hyperglycemic exposure could stimulate stress-mediated inflammatory mediators in cardiomyocytes in vitro. These stress-related changes in hyperglycemia-induced cardiomyocytes may further initiate an increase in injury markers which eventually could alter the epigenetic processes. Therefore, epigenetic and inflammatory mechanisms in conjunction with alterations in a downstream signaling pathway could have a direct effect on the functionality of the cardiomyocytes exposed to high glucose during short and long-term exposures.

## 1. Introduction

Diabetes mellitus is a disease characterized by the body’s inability to produce or properly respond to insulin which ultimately causes elevated blood glucose levels. Over time, high levels of blood glucose lead to micro- and macro-vascular damage to various organs of both Types 1 and 2 diabetic patients. Cardiomyopathy is one of the most life-threatening macro-vascular complications of the diabetes. Cardiomyopathy is defined as progressive heart disease that affects the heart muscle due to thickening, rigidity or enlargement of the muscles resulting in a dysfunctional heart [1]. As the heart grows weaker, patients may get afflicted with worsening complications such as heart failure, arrhythmias, or valve problems [1]. The human heart is made of various cells including cardiomyocytes (CMs), fibroblasts (FBs), endothelial cells (ECs), perivascular cells, smooth muscle cells and neuronal cells. However, approximately 70–85% of the human heart is composed of CMs [2]. In the current study, we examined the early signs of stress in CMs exposed to hyperglycemic conditions that may lead to alterations in cardiomyocyte signaling, changes in injury markers and downstream indicators of oxidative stress, as well as epigenetic modifications and alterations in inflammatory mediators.

Connexin 43 (Cx43) is a transmembrane gap junction protein located between adjacent cells that functions to coordinate the depolarization of cardiac muscle during contractions of the heart. Chronic hyperglycemic condition in diabetes may disrupt and change the intercellular communication due to alterations in Cx43. Troponins are a group of complex proteins that regulate muscular contraction in cardiac and skeletal muscles and used as a marker to detect cardiac damage. Troponins consist of three subunits (C, T, and I), localized in the myofibrils. Troponin I is a cardiac specific inhibitory isotype that determines myocardial impairment [3]. Hyperglycemic condition initiates reduced mitochondrial enzyme activity and an increase in reactive oxygen species (ROS) formation. Hyperglycemia-mediated rise in oxidative stress is also linked with increased advanced glycation end products (AGEs) formation along with the enhanced expression of the receptor for AGEs (RAGE). Increased RAGE expression causes activation of various cellular pathways along with release of high mobility group box 1 (HMGB1) that bind to RAGE and toll-like receptor 4 (TLR4) with high affinity eventually stimulating a host of proinflammatory events [4]. TLR4 is transmembrane pattern recognition receptor (PRR) family of proteins [5]. Previous studies have demonstrated that high glucose induces increase in TLR4 expression in cardiac cells in a dose- and time-dependent manner [6]. Heat shock proteins (HSP) are chaperone proteins that regulate intracellular signaling. Earlier studies have suggested the cardiac protective role of HSP60 in cardiomyocytes, and reduced expression of HSP60 under chronic hyperglycemic condition may contribute to the progression of diabetic cardiomyopathy [7,8,9]. Epigenetic mechanisms contribute to the continuous and potential molecular modifications including DNA methylation and histone modifications under hyperglycemic condition that may cause aberrant and long-lasting changes in cardiomyocytes pathophysiology. Histone 3 lysine 9 mono methylation (H3K9me1), a marker for post-translational histone modification has appeared to have a prospective role in the progression of cardiac complications in diabetes [10]. Histone methylation may lead to inflammation and damage to the cardiac cells, leading to cardiomyopathy [2]. High glucose may affect specific changes in histone marks at the promoter sites of genes encoding inflammatory cytokines in cardiomyocyte cell lines [11]. Dysregulation of epigenetic posttranscriptional modifications of histones in chromatin is thought to be associated with the pathology of many disease models, including CVD. Balance of histone acetyltransferases (HATs) and deacetylases (HDACs) control the histone lysine acetylation. HDAC activity also play a crucial role in the severity of the cardiac damage with respect to myocardial remodeling. HDAC2 expression is altered in hyperglycemic condition [12]. Sirtuin (SIRT) 1, a nicotinamide adenine dinucleotide (NAD)^+^-dependent class III histone deacetylase, has been reported to play a significant role in cellular metabolism, longevity as well as support against DNA damage [13,14]. In presence of stress, maintenance of NAD^+^ is challenged which could affect cellular functions such as insulin secretion and signaling [15]. SIRT1 has been linked with insulin sensitivity and glucose homeostasis and studies have shown that patients with diabetes had an 80% reduction in SIRT1 levels. The present study describes that increased oxidative stress induced by hyperglycemia on CMs could lead to altered levels of inflammatory and epigenetic markers which may ultimately cause cardiac dysfunction in patients with diabetes. 

## 2. Results

### 2.1. High Glucose Insult Results in Alterations in Connexin 43 and Troponin I Expression

**Figure 1:**
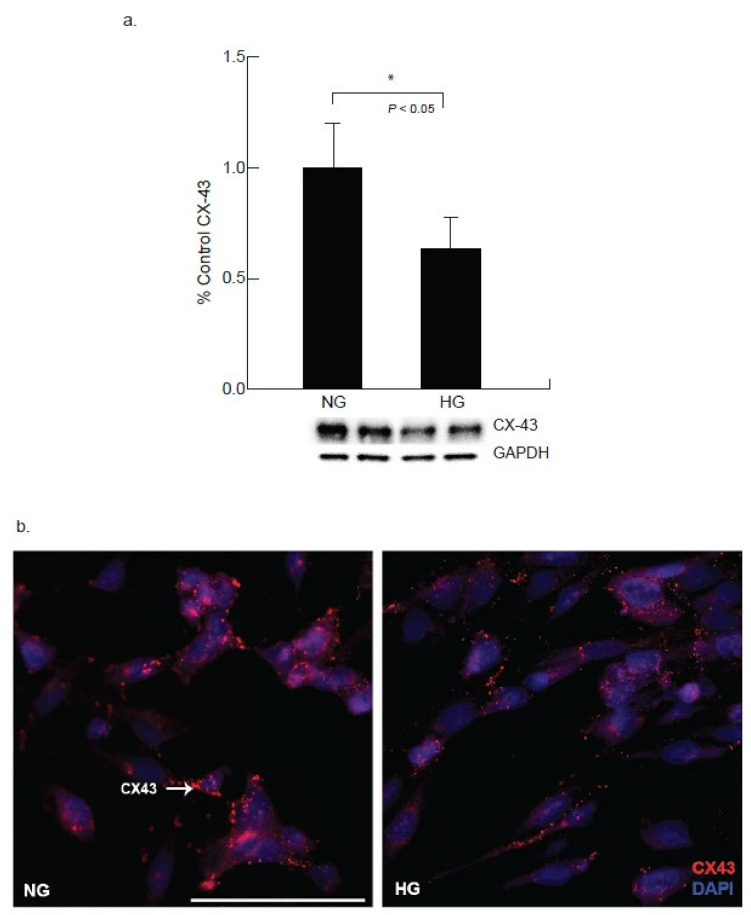
Hyperglycemic condition results in alterations in Connexin 43 expression in cardiomyocytes. Gap junction protein connexin 43 (Cx43) expression was assessed by Western blot analysis as well as by immunohistochemistry in cardiomycytes. (**a**). Western blot analysis demonstrated a decrease in CX43 expression in the hyperglycemia exposed cardiomyocytes compared to the normoglycemic condition (NG, * *p* < 0.05). (**b**) Immunocytochemical analysis also exhibited reduced CX43 expression (red) in CM under hyperglycemic condition compared to cardiomyocytes under normal glucose condition after 24 h of exposure. (Scale bar = 100 µm).

**Figure 2:**
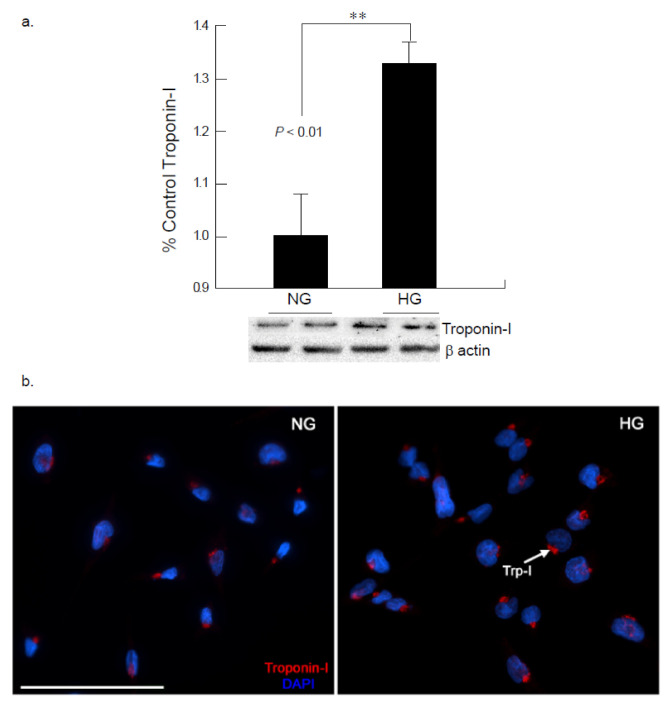
Hyperglycemia mediates increased Troponin I expression in the cardiomyocytes. (**a**) Western blot analysis of AC16 cardiomyocytes given hyperglycemic insult for 24 h showed an increase in the expression of Trp-I as compared to cells exposed to normal glucose (NG; ** *p* < 0.01). (**b**). Immunocytochemical analysis of cardiomyocytes exposed to high glucose (HG) for 24 h showed an increase in the expression of Troponin I (red). (Scale bar = 100 µm).

Connexin 43 (Cx43) is a gap junction protein that maintains normal heart functions by regulating ventricular contractions, though under stress the functionality is altered. Cx43 expression under hyperglycemic conditions (25 mM) exhibited significant down regulation at 24-h exposure. These observations were further confirmed via immunocytochemistry studies, where Cx43 expression was significantly downregulated in the presence of elevated glucose levels at 24 h on two separate experiments (Figure 1a,b). These findings were further analyzed for Troponin I (Trp-I) expression after 24 h hyperglycemic exposure to cardiomyocytes. Troponin I is required to regulate the contractions of the heart and skeletal muscles and upregulation of Trp-I demonstrates damage of the cardiomyocytes or heart muscles. Our study indicated a visible upregulation of Troponin I expression in CM cells exposed to the hyperglycemic conditions, when compared to the normo-glycemic condition (Figure 2).

### 2.2. Hyperglycemia Leads to Altered Signaling of HSP60

**Figure 3:**
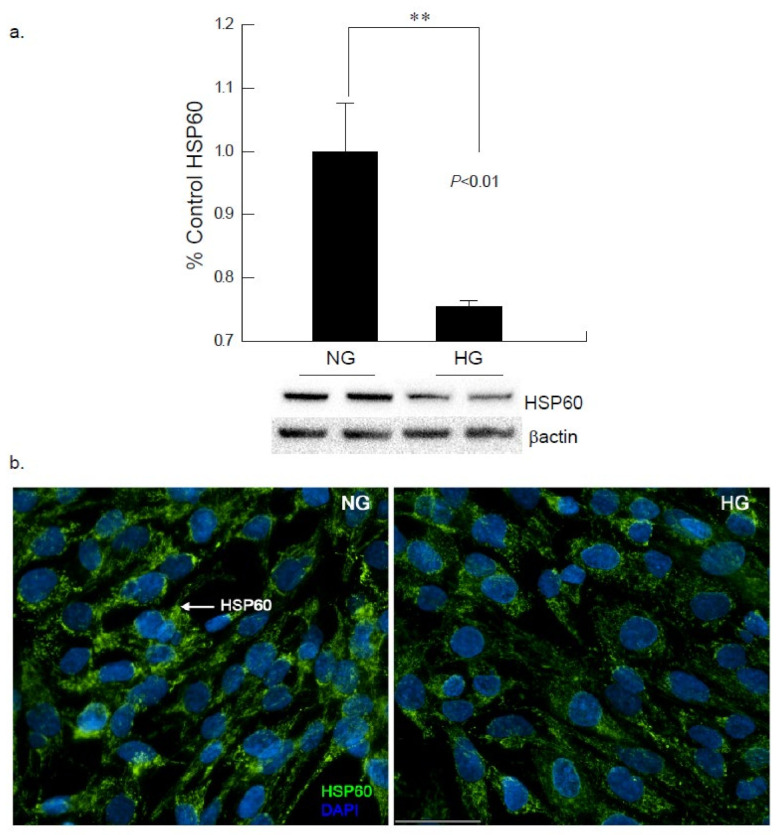
Hyperglycemic insult in cardiomyocytes reduces HSP60 expression. (**a**) Western blot analysis of AC16 cardiomyocytes following hyperglycemic insult for 48 h showed a decrease in the expression of HSP60 as compared to cells exposed to normal glucose (NG; ** *p* < 0.01). (**b**) Immunocytochemical analysis of cardiomyocytes exposed to high glucose (HG) for 48 h also confirmed the decrease in the expression of HSP60 (green). (Scale bar = 50 µm).

Heat shock protein 60 (HSP60) is a chaperone protein member of the HSP family which typically functions in mitochondria as well as cytosol, membrane and extracellular space, under stress, inflammation and immune mediated or other cellular events. Western blot analysis of HSP60 showed a downregulation of protein ability after a 48-h exposure to hyperglycemia, indicating that mechanisms may be altered as a result of the chronic stress (Figure 3). These findings were confirmed via immunocytochemistry studies, where a decrease in HSP60 was noted in the high glucose conditions as compared to the normal glucose conditions. Further analysis unveiled that heat shock protein 70 (HSP70), involved in inhibition of aggregation by binding to unfolded proteins by preventing further cellular damage, was significantly downregulated (*p < 0.05) after 24 and 48 h of exposure to an elevated glucose (25 mM) environment (Figure S1).

### 2.3. Hyperglycemia Induces Elevated Expression of HMGB1 and H3K9me1 in Cardiomyocyte

**Figure 4:**
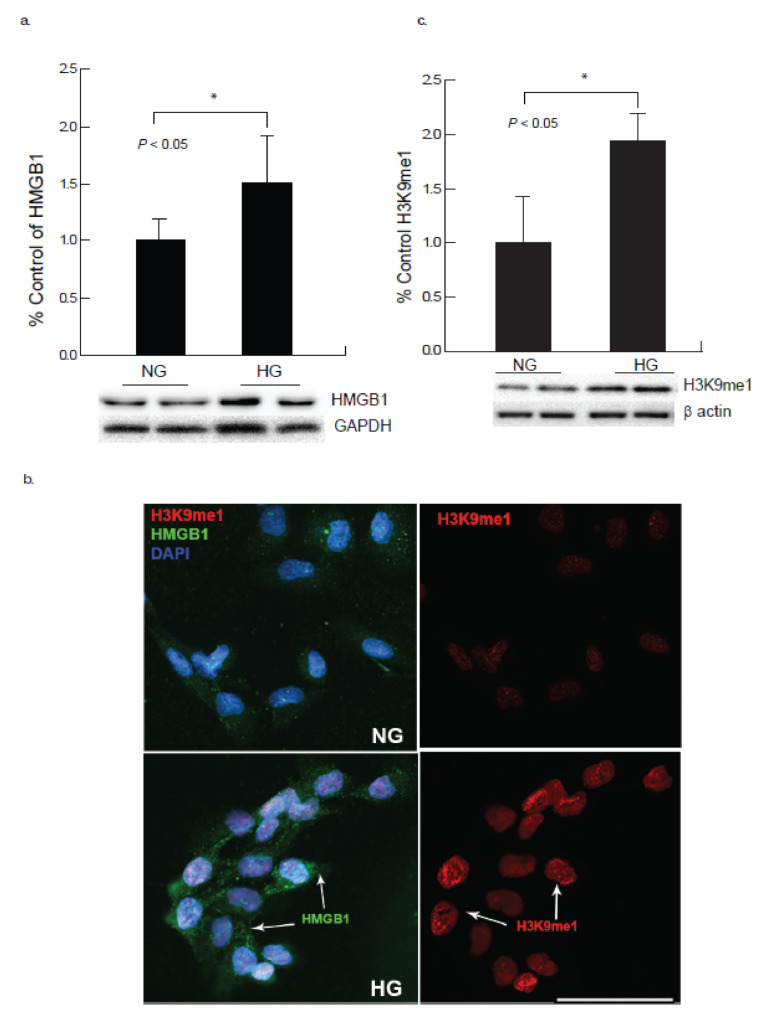
Hyperglycemia induces elevated expression of HMGB1 and H3K9me1 in cardiomyocyte. AC-16 cardiomyocytes revealed that stress mediated high glucose exposure increased expression of histone 3 lysine 9 mono-methylation (H3K9me1) as well as inflammatory mediator HMGB1 (high mobility group box 1). (**a**) Western blot analysis showed an increase in HMGB1 after 24 h of exposure to high glucose (HG) in comparison to normal glucose conditions (NG; * *p* < 0.05). (**b**) Immunocytochemical analysis of cardiomyocytes exposed to high glucose for 24 h showed an increase in the expression of H3K9me1 (red) along with increased inflammatory mediator HMGB1 (green). (Scale bar = 100 µm). (**c**) Western blot analysis showed an increase in H3K9me1 after 24 h of exposure to high glucose in comparison to normal glucose conditions (NG; * *p* < 0.05).

**Figure 5:**
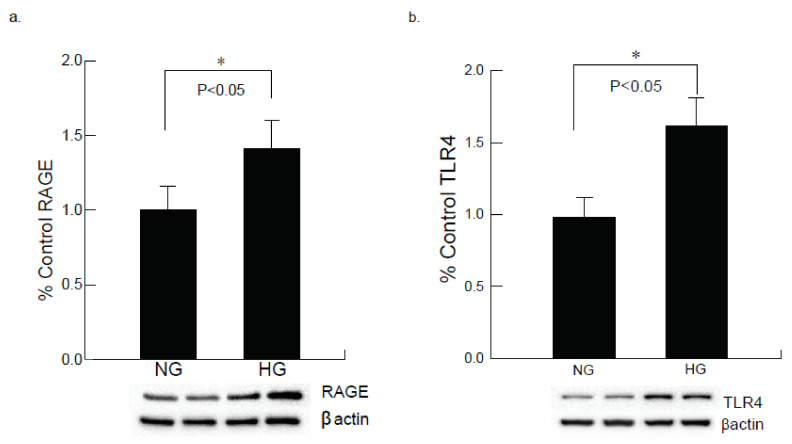
Hyperglycemic exposure exhibits altered levels of RAGE and TLR4 in AC-16 cardiomyocytes. (**a**) Increased RAGE expression was observed under the hyperglycemic condition in cardiomyocytes compared to normo-glycemic condition (* *p* < 0.05). (**b**) Western blot analysis of AC16 cardiomyocytes under hyperglycemic conditions for 24 h showed a statistically significant increase in the expression of TLR4 as compared to cells exposed to normal glucose (* *p* < 0.05).

HMGB1 is a non-chromosomal DNA-binding protein, which may release from cardiomyocytes under certain physiological conditions in response to stress. The immunocytochemistry and Western blot studies demonstrated an upregulation of HMGB1 (Figure 4a,b) along with its receptors RAGE and TLR4 (Figure 5a,b), that may lead to damage of CM cells as indicated by the upregulation of other stress-induced markers after 24 h of hyperglycemic exposure. 

Epigenetic modifications play critical part in cardiac dysfunction and not many studies demonstrated the role of epigenetic processes and cardiac dysfunction under hyperglycemic milieu. High glucose conditions may modify gene transcription by changing the post-translational modifications of histone protein. Our study showed a significant increase of histone mono-methylation at lysine 9 (H3K9me1) in CM cells in the presence of high glucose (Figure 4b,c) as demonstrated by immunocytochemistry and Western blot studies.

### 2.4. AC-16 Cells under Hyperglycemic Conditions Upregulates RAGE and TLR4 Expression

Following 24-h exposure to high glucose, the cardiomyocytes exhibited an increase in oxidative stress marker, receptor for advanced glycation end products (RAGE) and inflammatory mediator toll-like receptor 4 (TLR4) expression as evaluated by western blot analysis (Figure 5a,b). The RAGE is expressed in the cardiomyocytes, and an increase in RAGE expression suggests injuries to the CM cells due to hyperglycemic condition. Both RAGE and TLR4 bind to its ligand HMGB1.

### 2.5. Hyperglycemia Modifies Chemokine Receptor CXCR4 Expression in Cardiomyocytes

**Figure 6:**
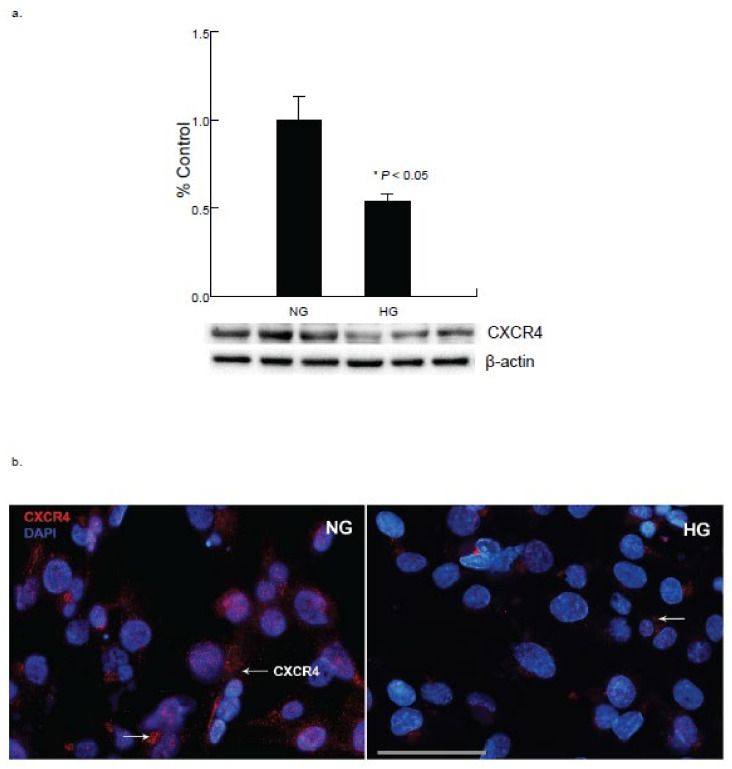
Hyperglycemia alters chemokine receptor CXCR4 expression in cardiomyocytes. (**a**) Western blot analysis of cardiomyocytes exposed to hyperglycemic condition for 48 h showed reduction in the expression of CXCR4 (* *p* < 0.05). (**b**) Immunocytochemical analysis of cardiomyocytes exposed to high glucose (HG) for 48 h showed decrease in the expression of CXCR4 (red). (Scale bar= 100 µm).

To understand the underlying mechanisms and the role of inflammatory mediators in cardiac impairment, the CXC chemokine receptor 4 (CXCR4), a G protein-coupled receptor (GPCR) was evaluated. Western blot analysis of the cardiomyocytes demonstrated a significant decrease in the CXCR4 expression at 48 h of exposure under hyperglycemic condition compared to the normo-glycemic condition (Figure 6), which further confirmed its possible role in prolonged inflammation. The decrease in the CXCR4 expression by long-term exposure to hyperglycemia induces damage to CM cells. 

### 2.6. Hyperglycemia Leads to Increased HDAC2 Expression in Cardiomyocyte

**Figure 7:**
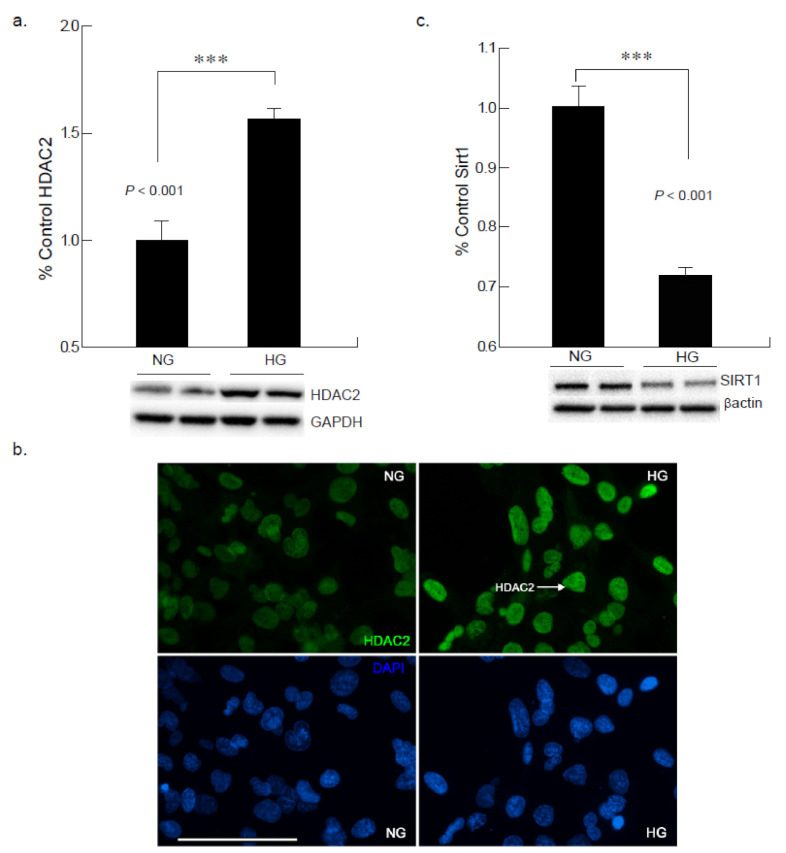
Hyperglycemic exposure exhibits alterations in epigenetic modifications with increased expression of HDAC2 and decreased expression of SIRT1 in cardiomyocytes in vitro. (**a**) Western blot analysis showed an increase in HDAC2 after 24 h of exposure to high glucose (HG) in comparison to normal glucose conditions (NG; *** *p* < 0.001). (**b**) Immunocytochemical analysis of cardiomyocytes exposed to high glucose for 24 h showed an increase in the expression of HDAC2 (green) (Scale bar= 100 µm). (**c**) Cardiomyocytes exposed to high glucose for 48 h showed reduction in SIRT1 expression (*** *p* < 0.001).

To investigate the potential role of epigenetic modifications of class I histone deacetylases in cardiac cells under hyperglycemic condition, we analyzed the expression of HDAC2 in AC-16 cells that are exposed to 25 mM of glucose for 24 and 48 h. Western blot and immunocytochemistry were performed to analyze the expression of HDAC2 in cardiomyocytes under normoglycemic and hyperglycemic conditions. The results indicated that the protein levels of HDAC2 were markedly elevated in hyperglycemic cardiomyocytes compared the normoglycemic cardiomyocytes after 24 h of high glucose exposure (Figure 7a,b).

### 2.7. Hyperglycemic Condition Downregulates Sirtuin-1 Expression in CM

To define whether physiologically-relevant hyperglycemic conditions could further affect class III histone deacetylase, SIRT1 expression, AC16 CM cells were exposed to high glucose (25 mM) media; 48 h of exposure exhibited a decreased expression in SIRT1 levels. SIRT1 is an NAD+ dependent deacetylase which is important for metabolic control; when exposed to hyperglycemic induced stress, a shift in the NADH/NAD+ratio has been observed. Given the possible association between hyperglycemia and inflammation, the results confirm an inverse relationship between SIRT1 expression and hyperglycemic condition. Following a 48-h incubation period in elevated glucose, a significant decrease (***p < 0.001) of SIRT1 was noted (Figure 7c).

## 3. Discussion

The purpose of this study was to distinguish if alterations in glucose levels in AC16 cardiomyocyte cells could result in the activation of epigenetic and inflammatory mechanisms that may affect post-translational modification of oxidative stress mediators. Hyperglycemia increased the expression levels of oxidative stress mediators in AC16 cardiomyocytes after 24- and 48-h of exposure to high glucose.

Connexin 43 (Cx43), a gap junction protein, located between cardiomyocyte cells was noted to be down-regulated in the presence of hyperglycemia in the present study. The development and progression of diabetic cardiomyopathy is associated with gap junction activity for intercellular communication [16]. The effect of hyperglycemic conditions on the expression of cardiomyocyte-specific connexin 43 was examined to delineate the activity of the primary gap junction protein for intercellular communication. The results reveal that high glucose concentrations inhibited gap junction activity by reducing Cx43 synthesis in AC16 cardiomyocytes. Degradation of Cx43 will decrease the gap junction activity which may also decrease the conductivity in the cardiomyocytes. Impaired intercellular communication may impact to further changes in cardiac damage markers [17]. In conjunction to this, increased Troponin I expression seen in the AC16 cell line further promotes the concept that the cardiomyocytes may demonstrate injury which is secondary to chronic hyperglycemic stress. The troponin complex is often used to measure cardiac damage in the presence of prolonged stress [18]. More specifically, Troponin I is released in response to stress-induced permanent damage to the cardiac structure [19]. The alterations in Cx43 and Troponin I expressions as evaluated by western blot and immunocytochemical studies after 24-h exposure suggest the impaired function of the cardiomyocytes under hyperglycemic stress.

Lastly, it was noted via immunocytochemical analysis that the expression of HMGB1 and histone markers were upregulated, which may demonstrate cardiomyocyte apoptosis. HMGB1 is a stress related mediator that plays a pro-inflammatory role when released [20]. This in turn could lead to upregulated immune responses and in turn initiation of apoptotic pathways as the development of cardiomyopathy under hyperglycemia. H3K9me1 and HDAC2 in turn have the role of silencing transcription of genes. Since both followed an upregulated trend at 24 h, it may be assumed that the transcription of cardiomyocytes is being repressed as a result of the pro-inflammatory mechanisms initiated by HMGB1. Prior research has made it evident that inhibiting HDACs promotes healthy cell development and function, which in turn reduces complications associated with elevated blood glucose levels [21]. SIRT1 activation is induced by increased ionized NAD, and conversely a shift in the NADH/NAD+ ratio, commonly observed in hyperglycemia, decreases SIRT1 expression, potentially leading to detrimental effects in the cell.

In the present study a downregulation in epigenetic marker SIRT1 was apparent in the cells that were exposed to hyperglycemia after 48-h exposure. SIRT1 is an NAD+-dependent deacetylase involved in metabolic control [15]. The most probable explanation as to why behind these results could be that under hyperglycemic-induced stress, maintenance of NAD+ is challenged. This, then, reduces the pro-survival effects such as: clearance of toxic aggregates, activation of proteolytic enzymes, enhanced expression of chaperone proteins, and inflammation reduction associated with SIRT1 [22]. This suggests that a crosstalk occurring between epigenetic mechanisms and the inflammatory pathway may show inverse relationship as evident by downregulation of SIRT1 expression [23]. Additionally, there was also evidence for a significant decrease in HSP60 expression and a downward trend for HSP70 expression noted throughout the study [8]. The HSP families are tasked with insuring proper folding of proteins and preventing damaged proteins from continuation of folding [24]. Given that SIRT1 was downregulated after 48-h high glucose exposure, which supports that there is communication between the epigenetic mechanisms and the stress-mediation mechanisms in response to increased oxidative stress [25]. This also substantiates that there is possible crosstalk between the epigenetic mechanisms and inflammatory pathways.

It is important to understand the unescapable role of epigenetic processes that are implicated in the development of diabetes complications. The relationship between microvascular and macrovascular complications of diabetes and epigenetic processes has been commonly investigated [26]. Whereas, epigenetic dysregulation towards cardiac atrophy in diabetes has not been investigated thoroughly. The two most commonly studied epigenetic modifications, DNA methylation and the post-translational modification of histone proteins have shown to play key roles in cardiac development as well as cardiac dysfunction [27].

This study suggests that targeting epigenetic markers and stress-mediated responders may impact future approaches in the treatment of cardiomyopathy that has been developed secondary to hyperglycemia. Understanding the crosstalk between the inflammatory mechanisms and epigenetic markers could help researchers and clinicians design enhanced testing methods which could in turn translate into better detection methods and allow for earlier intervention.

## 4. Materials and Methods

### 4.1. AC16 Human Cardiomyocyte Cell Line Culture

AC16 human cardiomyocyte cell line (MilliporeSigma, St. Louis, MO, USA; cat no. SCC109) were cultured in Dulbecco’s Modified Eagle’s Medium/Nutrient Mixture F-12 Ham (DME/F-12) supplemented with 10% fetal bovine serum (FBS). This cell line was originally derived from the fusion of primary adult human ventricular heart tissue cells with SV40 transformed, uridine auxotroph human fibroblasts, lacking mitochondrial DNA. Following the use of a uridine-free medium as a selection for the removal of unfused fibroblasts, the remaining fused cells were sub-cloned and screened for the presence of SV40 large T-ag, β-myosin heavy chain (βMHC) and connexin 43 (Cx43) [28]. The cells were plated with approximately 40% confluency at 24-h and 30% confluency for 48-h experiments in 12-well plates for high glucose exposure assessments. When the cells reached 70-80% confluency, the wells were divided evenly into a control group containing 5 mM of glucose and an experimental group exposed to an additional 25 mM of glucose for hyperglycemic condition.

### 4.2. Protein Extraction and Western Blot Analysis

Following the experimental high glucose exposure period of 24 and 48 h respectively, the AC16 cells were harvested using lysis buffer with protease and phosphatase inhibitors and processed for western blot as described in our previous work [29,30]. The primary antibodies for RAGE, CX43, HMGB1, SIRT1 (Cell Signaling, Danvers, MA, USA), CXCR4, Trp-I, TLR4, (Thermo Scientific, Waltham, MA, USA), H3K9me1 (Epigentek, Farmingdale, NY, USA), HSP60 (MilliporeSigma, St. Louis, MO, USA) and secondary antibody anti-rabbit IgG or anti-mouse IgG (1:5000 Amersham, Piscataway, NJ, USA) were used according to the protocol and imaged with ECL (Pierce, Rockford, IL, USA). β-actin (1:2000; MilliporeSigma, St. Louis, MO, USA) was used as a loading control and the data were normalized with the respective level of β-actin using an image analysis software (ChemiDoc XRS System, Bio-Rad Laboratories, Hercules, CA, USA) to determine the intensity of each band (n = 3–4 wells per group), the data were further analyzed to assess the percent of control.

### 4.3. Immunocytochemistry

AC16 cells cultured in coverslips, were rinsed in 1X phosphate buffered saline (PBS) and subsequently fixed in methanol for 15 min. The coverslips were then blocked at room temperature for 60 min, were washed once and incubated with either of the primary antibodies CX43, HMGB1 (1:400; Cell Signaling, Danvers, MA, USA) or CXCR4, Troponin I (1:500; Thermo-Fisher Scientific, Waltham, MA, USA), H3K9me1 (Epigentek, Farmingdale, NY, USA), for overnight at 4°C followed by three washes. Alexa Fluor 594 goat anti-rabbit IgG and 488 goat anti-mouse IgG (1:2000; Thermo-Fisher Scientific, Waltham, MA, USA) used as secondary antibodies and were incubated for one hour at room temperature. The cells were rinsed in 1X PBS for 3 times and stained with Hoechst solution (1:50,000). The coverslips were rinsed in 1X PBS for three 5-min intervals, mounted on glass slides using of Fluoromount G mounting media (Electron Microscopy Sciences, Fort Washington, PA, USA). CM grown on coverslips were immunostained with HMGB1, H3K9me1, CXCR4, CX-43 or Trp-I and imaged to evaluate for changes in intensity to identify the effects of the hyperglycemia. The images were acquired with 20X objective using a Nikon Eclipse Ni-E microscope (Nikon Instruments Inc., Melville, NY, USA).

### 4.4. Statistical Analysis

Normoglycemic and hyperglycemic samples were evaluated to analyze the changes in protein expression. A one-way analysis of variance (ANOVA, parametric) was conducted for the group comparisons using the Bonferroni correction for multiple post hoc analyses. All the statistical analysis was performed using SPSS software (Systat version 13.0, SPSS Inc., Chicago, IL, USA), and a *p*-value ≤ 0.05 was considered to be statistically significant. The results were demonstrated as mean ± SEM.

## Data Availability

All data generated or analyzed during the current study are included in this published article.

## Supplementary Materials

The following are available online at https://www.mdpi.com/article/10.3390/ijms221910802/s1. FigureS1. Hyperglycemic insult in cardiomyocytes reduces HS70 expression. (a) Western blot analysis of AC16 cardiomyocytes following hyperglycemic insult for 48 hours showed a decrease in the expression of HSP70 as compared to cells exposed to normal glucose (NG* *p* < 0.05).

## Abbreviations

CXCR4	chemokine C-X-C motif receptor 4
RAGE	receptor for advanced glycation end products
Toll-like receptor 4	TLR4
CX43	Connexin-43
Trp-I:	Troponin I
Sirtuin 1	SIRT1
Histone deacetylase 2	HDAC2
Heat shock protein 60	HSP60
Histone 3 lysine 9 mono-methylation	H3K9me1
High mobility group box 1	HMGB1
