# Supplementary material for: Changes in Stress-Mediated Markers in a Human Cardiomyocyte Cell Line under Hyperglycemia
Source: Int J Mol Sci. 2021 Oct 7;22(19):10802. doi: 10.3390/ijms221910802 (PMC8509354; doi:10.3390/ijms221910802)
Supplement: Supplementary file 1 [file ijms-22-10802-s001.zip › ijms-1392834-supplementary.pdf]

Suppl. Fig.1.

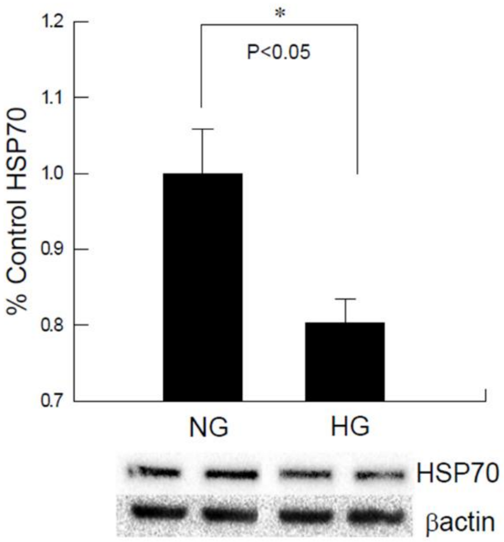

Suppl. Fig.1. Hyperglycemic insult in cardiomyocytes reduces HS70 expression. (a) Western blot analysis of AC16 cardiomyocytes following hyperglycemic insult for 48 hours showed a decrease in the expression of HSP70 as compared to cells exposed to normal glucose (NG).
